# Clinical recommendations for chronic musculoskeletal pain in South African primary health care

**DOI:** 10.4102/phcfm.v15i1.3929

**Published:** 2023-04-25

**Authors:** Dawn V. Ernstzen, Romy Parker, Tasleem Ras, Klaus Von Pressentin, Quinette A. Louw

**Affiliations:** 1 Division of Physiotherapy, Department of Health and Rehabilitation Sciences, Faculty of Medicine and Health Sciences, Stellenbosch University, Cape Town, South Africa; 2 Pain Management Unit, Department of Anaesthesia and Perioperative Medicine, Faculty of Health Sciences, University of Cape Town, Cape Town, South Africa; 3 Groote Schuur Hospital, Cape Town, South Africa; 4 Division of Family Medicine, Department of Family, Community and Emergency Care, Faculty of Health Sciences, University of Cape Town, Cape Town, South Africa

**Keywords:** chronic musculoskeletal pain, clinical practice guidelines, consensus methods, primary health care, multidisciplinary, contextually relevant

## Abstract

**Background:**

Chronic musculoskeletal pain (CMSP) is prevalent globally and places a significant burden on individuals, healthcare systems and economies. Contextually appropriate clinical practice guidelines (CPGs) on CMSP are advocated to translate evidence into practice.

**Aim:**

This study aimed to investigate the applicability and feasibility of evidence-based CPG recommendations for adults with CMSP in the primary health care (PHC) sector of South Africa (SA).

**Setting:**

The PHC sector in South Africa (SA).

**Methods:**

Consensus methodology was used, comprising two online Delphi rounds and a consensus meeting. A multidisciplinary panel of local healthcare professionals involved in CMSP management was purposefully sampled and invited to participate. The first Delphi survey considered 43 recommendations. In the consensus meeting, the results of the first Delphi round were discussed. The second Delphi round reconsidered the recommendations with no consensus.

**Results:**

Seventeen experts participated in the first Delphi round, 13 in the consensus meeting and 14 in the second Delphi round. In Delphi round two, 40 recommendations were endorsed, three were not endorsed and an additional recommendation was added.

**Conclusion:**

A multidisciplinary panel endorsed 41 multimodal clinical recommendations as applicable and feasible for the PHC of adults with CMSP, in SA. Although certain recommendations were endorsed, they may not be readily implementable in SA because of context factors.

**Contribution:**

The study forms the basis of a model of care for contextually relevant PHC of CMSP. Future research should explore factors that could influence the uptake of the recommendations into practice to optimise chronic pain care in SA.

## Introduction

Chronic pain and disability are a global and local South African (SA) concern. The pooled prevalence of chronic pain in developing countries is 18% (95% confidence interval: 10% – 29%), (range 13% and 51%),^1^ with South Africans having a similar prevalence of 20% in women and 18% in men.^2^ In addition, one in three South Africans over the age of 65 years suffers from chronic pain.^2^ Chronic pain is defined as persistent pain on most days, lasting longer than 3 months and persists beyond the expected healing time. Chronic musculoskeletal pain (CMSP) comprises pain associated with bone, joints, muscles and related soft tissue.^3,4^ The World Health Organization (WHO) in collaboration with International Association for the Study of Pain (IASP) recently endorsed the International Classification of Diseases, 11th edition (ICD-11), which includes a classification of chronic pain. The classification comprises chronic primary pain, chronic cancer pain, chronic post-surgical pain, chronic neuropathic pain, chronic headache and orofacial pain, chronic visceral pain and CMSP.^4^ The classification also acknowledges pain mechanisms within chronic pain classification as nociplastic, nociceptive and neuropathic pain.^3^

Chronic musculoskeletal pain is a multidimensional phenomenon, comprising interactions between biological, psychological, behavioural, social and environmental factors.^3,5^ Therefore, inter-disciplinary and multi-disciplinary care, inclusive of pharmacological and non-pharmacological management options, are essential for the holistic management of CMSP. While multiple therapies for CMSP are available and indicated, there exists uncertainty in the applicability of management options when considering different healthcare contexts. Clinical practice guidelines (CPGs) are particularly valuable where uncertainties about management options exist and where scientific evidence for an intervention is scarce.^6^ Clinical practice guidelines for CMSP have the potential to optimise chronic pain management and health systems outcomes, which is important goals in a country such as SA, with a transforming healthcare system.

South Africa is an upper-middle-income country,^7^ which must address the complex needs of people living in upper- middle- or lower-income settings in nine different provinces. Healthcare is provided through public and private healthcare systems, and there are many inequalities in healthcare access and resources between and within these systems.^8^ Vulnerable and remote communities are particularly affected by these disparities. As part of healthcare transformation, the SA government developed the National Health Insurance (NHI), which aims to provide equitable healthcare access to all South Africans. The NHI of South Africa White Paper (DoH)^9^ proposes CPGs to guide the delivery of evidence-based and cost-effective health services. The development of a new CPG can be resource and skill-intensive, and therefore existing good-quality CPGs can be adopted, adapted or contextualised to be implemented. The use of alternative approaches to CPG development enables resources to be channelled towards CPG implementation. Furthermore, CPGs from high-income countries may not be appropriate in resource-constrained environments because of differences in policy and legislation, organisational context, health system resources, human resources and capacity, access to healthcare, infrastructure, and patient resources and needs.^10^ Contextually relevant CPGs may play an important role in the uptake of evidence-based practice as part of healthcare restructuring. However, according to a recent landscape analysis of CPGs for the SA context, no CPG for CMSP management in SA exists.^11^ The existence of good quality CPGs on CMSP, and the range of contextual factors that may influence pain management in SA, indicated the need for alternative CPG development. The implementation of a contextually relevant CPG for CMSP that is acceptable and feasible for the SA context has the potential to support local decision-making and person-centred care in primary health care (PHC) because the majority of chronic pain conditions are managed in PHC.^12,13^

This study therefore focusses on addressing the knowledge gap on the acceptability and feasibility of existing CPG recommendations for implementation in the local, South African PHC context. The existing CPG recommendations were extracted from high-quality CPGs.^14,15^ The information on acceptability and feasibility would provide information on which of the clinical recommendations could be adopted, and which would need to be adapted or contextualised.^10^ Additionally, the approach would provide contextual information for the modification and implementation of recommendations. Adoption of recommendations implies accepting the recommendation without change, adaptation means changing the recommendation to suit local needs and contextualisation refers to adding context points to the recommendation to optimise implementation.^10^ This study evaluates the applicability and feasibility of internationally developed evidence-based clinical recommendations and endorses them for the management of adults with CMSP in South Africa primary health care (SA PHC). The secondary objectives were to identify possible contextual factors that may influence the uptake of the recommendations and customise the recommendations for implementation in the intended context based on the contextual information.

## Research methods and design

### Study design

*A priori* consensus methodology involving a group of local experts was used,^16^ comprising two Delphi study rounds and a face-to-face consensus meeting. The Delphi process aims to structure group communication processes efficiently to develop a systematic consensus of opinion regarding specific circumstances.^16,17^ Consensus methodologies are often used in decision-making regarding the appropriateness of clinical recommendations.^16,18^ The consensus meeting allowed interactive discussion about key issues, including the implementation of recommendations. The study formed part of a broader project to develop tailored guidance for the PHC of CMSP in the SA context. The project phases comprised an analysis of context, a systematic review of CPGs, evidence synthesis, contextualisation (Delphi study) and an external review.

### Study population and sampling

The study population comprised healthcare professionals involved in chronic pain management in the SA context. A multidisciplinary group of healthcare professionals was identified through purposive sampling and invited to participate. Panellists participating in consensus processes are typically individuals who have practical experience and interest in the topic being investigated.^16^ The key sampling criteria considered the diversity of opinions and experiences to define the expert panel as follows:

- Experience, expertise and interest in CMSP management and/or
- Work experience in any of the different healthcare settings or sectors (public PHC, private practice, academic institutions, and professional organisations); and/or
- Different clinical disciplines (medical doctors, clinical nurse practitioners, pharmacologists, physiotherapists, occupational therapists, psychologists, managers, researchers); and/or
- Expertise in CPG writing and use; and
- At least 3 years’ experience in the field.

As it is important to include patients’ views in guideline development, patient views were considered in a separate research phase, as part of implementation strategies for the recommendations.^19^

Potential participants were identified by the authors through contributions in conferences focussing on pain management and public health, publications and public healthcare sector involvement. These potential participants were then requested to suggest other potential participants who may fit the inclusion criteria.^18^ Twenty-six potential participants were identified and invited to participate via email and/or by personal discussion with the principal investigator. The purpose of the study, the process of developing a clinical recommendations, the consensus process, informed consent and the conflict of interest were explained. The aim was to recruit between 10 and 18 participants, as this number is recommended for consensus generation and group dynamics.^20^

### Development of evidence-based recommendations prior to consensus study

Prior to conducting the consensus study, we conducted a systematic review to identify high-quality CPGs for PHC of CMSP^14^ and we extracted and synthesised 43 clinical recommendations from them.^15^ Clinical practice guidelines were classified as high quality when they achieved a median score of 50% or more for the domain rigour of development on the Appraisal of Guidelines Research and Evaluation, Version II (AGREE II).^21^ A summary of the systematic review findings is available in Table 1. The consensus approach described in this study utilised the list of 43 extracted recommendations to ascertain consensus about the applicability and feasibility of each recommendation for the SA PHC context.

**TABLE 1:** Summary of systematic review findings.

Topic	Systematic review: Quality analysis	Systematic review: Content synthesis
Author	Ernstzen et al.^14^	Ernstzen et al.^15^
Study focus	Meta-synthesis of evidence-based CPGs for the management of CMSP in adults in PHC settings	Content analysis and synthesis of recommendations from high-quality CPGs for the PHC of adults with CMSP
Key findings	Twelve CPGs were eligible for inclusion. Six CPGs were considered high-quality based on their AGREE II scores. The CPGs varied in scope and methodological quality	A total of 156 recommendations were extracted from the six high-quality CPGs, which were condensed and synthesised into 43 multimodal recommendations
Key recommendations	Future CPGs for CMSP should include patient preferences and values and consider factors that impact the applicability of recommendations. Because several high-quality CPG on the topic exists, existing CPGs may be tailored to suit local needs to optimise implementation	The multimodal list of evidence-based clinical recommendations for the PHC of CMSP may be used to prioritise and contextualise key recommendations that would be impactful to address the burden of CMSP, particularly in LMICs. The core list can be used to monitor and evaluate care processes and inform future research

AGREE II, Appraisal of Guidelines Research and Evaluation, Version II; CMSP, chronic musculoskeletal pain; CPG, clinical practice guideline; PHC, primary health care; LMIC, low- and middle-income countries.

Note: Please see the full reference list of the article, Ernstzen DV, Parker R, Ras T, Von Pressentin K, Louw, QA. Clinical recommendations for chronic musculoskeletal pain in South African primary health care. Afr J Prm Health Care Fam Med. 2023;15(1), a3929. https://doi.org/10.4102/phcfm.v15i1.3929, for more information.

### Data collection

Participants completed an online Delphi survey via Stellenbosch University surveys (SUNsurvey), using an emailed survey link. The survey provided a short introduction about the study, a request for informed consent and instructions on how to complete the survey. The participants were presented with 43 recommendations, accompanied by the strength of the body of evidence (SoBE) for each recommendation. A five-point Likert scale (strongly agree [1], partly agree [2], undecided [3], partly disagree [4] and strongly disagree [5]) was used for voting. A non-applicable (n/a) key, which was considered a missing data point, was available if a statement was outside the scope of expertise of the participant. The survey requested participants to consider the applicability and feasibility of the clinical recommendation to endorse the recommendation for the SA context. Applicability or feasibility was defined as the ability to put a recommendation into practice when considering the barriers and facilitators to implementation. Applicability or feasibility considers the population in the intended setting, as well as the knowledge, skill, staff, time frames, equipment and resources that influence applicability.^22^ The participants were able to add a comment per recommendation to elaborate on their vote. The survey was open for 2 weeks. Two reminders were sent before the closing date of the survey. Prior to the consensus meeting, all responses were downloaded from SUNsurvey, de-identified and summarised by the primary investigator (PI) and analysed by the research team.

Two weeks after the survey closed, a face-to-face consensus meeting was held. The purpose was to present the results of the first Delphi round and to discuss recommendations for which consensus was not reached after the Delphi round (i.e. recommendations for which the Likert responses were 3 or above; or no consensus). The meeting was audiotaped, and a summary of discussions and decisions was made by the PI. After the meeting, the research team revised the wording of the recommendations to ensure that they incorporated the panel comments and were grammatically correct.

The second-round online survey was administered 1 week after the consensus meeting. Participants could vote for the adapted or contextualised recommendations using the same Likert scale. The survey was open for 3 weeks. Two reminders were sent before the closing date of the survey.

The steps leading to the list of recommendations are summarised as follows:

- Step 1: Conduct a systematic review to identify high-quality CPGs on the PHC of CMSP^14^
- Step 2: Extract and synthesise a core list of recommendations for PHC of CMSP from existing high-quality CPGs^15^
- Step 3: Panel evaluate and endorse or reject each recommendation for its applicability and feasibility in the SA PHC context (Delphi round 1)
- Step 4: Consensus meeting to discuss Delphi round 1 results
- Step 5: Panel re-evaluate and endorse or reject adapted or contextualised recommendations for their applicability and feasibility in SA PHC context (Delphi round 2)
- Step 6: Panel produce a list of recommendations endorsed for the SA PHC.

### Data management and analysis

The data were extracted from the online survey into an Excel spreadsheet. An explicit method for the combination of results for the two Delphi rounds was used.^16^ The median was used as a measure of central tendency to represent the combined opinion of the participants.^16,23^ The interquartile range (IQR) was used to indicate the level of dispersion from the median.^17^ Consensus could indicate the combined agreement or disagreement with a recommendation. Three criteria for consensus were applied. Firstly, an IQR of 1 or less was defined as consensus as this is a suitable consensus indicator for a five-unit scale.^17^ Secondly, the study aimed to reach a consensus for 95% of items before terminating rounds (i.e. 39 out of the 43 recommendations). It was estimated that two to three rounds would be sufficient because round one started with predetermined evidence statements, instead of generating ideas (as for a classic Delphi study).^20^ Each comment that participants added within the survey or consensus discussion remained linked to its recommendation and was analysed narratively. All recommendations on pharmacological management were externally reviewed by a pharmacologist, as we were unable to recruit a pharmacologist on the expert panel.

### Quality assurance measures

For the Delphi rounds, voting was independent and confidential. The panel members signed a conflict-of-interest document to declare any actual or potential financial, professional affiliation and intellectual conflicts of interest that may have a direct influence on the content of the recommendations. The researchers were committed to neutrality in the process. In the consensus meeting, the PI facilitated the discussion, and the research team provided input to the rest of the group. This process is inherent to a consensus meeting (group decision-making). The researchers’ engagement in the consensus meeting was grounded in the evidence statements and making sure that each member contributed equally. Only D.E. and Q.L. were involved in the data analysis and the rest of the research team contributed to the interpretation of the results. The researchers also completed a conflict of interest declaration. An independent research assistant (a social anthropologist) attended the consensus meeting and audited the data.

### Ethical considerations

The study protocol was approved by the Stellenbosch University Health Research Ethics Committee (HREC), SA. Electronic (tick box) informed consent was obtained on the online Delphi questionnaire and written informed consent and conflict of interest declarations was obtained at the consensus meeting. The survey enabled confidential responses and anonymous extraction of data. Because of the nature of the in-person consensus meeting, participants were aware of each other’s participation but were requested to uphold the confidentiality of the information shared. Participants were aware that they could exit the study at any point in time. The recommendations for the Conducting and REporting of DElphi Studies (CREDES)^18^ were used for reporting.

## Results

The researchers invited 26 potential participants and 17 of them participated in the Delphi round one, 14 participated in the consensus meeting and 13 in the second Delphi round. The reasons for non-participation were time constraints and geographical distance (for the consensus meeting). Table 2 provides an overview of the participants’ characteristics. Physiotherapists were the largest professional group at the consensus meeting. Thirteen of the round one participants were from the Western Cape province.

**TABLE 2:** Characteristics of participants in the consensus development.

Variable	Delphi 1 (*N* = 17)		Meeting (*N* = 13)		Delphi 2 (*N* = 14)	
	*n*	%	*n*	%	*n*	%
**Gender**						
Women	13	76	12	92	12	86
Men	4	24	1	8	2	14
**Occupation**						
Clinical nurse practitioner	3	18	1	8	3	21
Medical doctor	4	24	1	8	2	14
Occupational therapist	2	12	1	8	2	14
Physiotherapist	5	29	9	69	5	36
Psychologist	3	18	0	0	2	14
Social anthropologist	-	-	1	8	-	-
**Years involved with the management of CMSP†**						
0–3 years	1	6	-	-	-	-
4–6 years	2	12	-	-	-	-
7–10 years	4	24	-	-	-	-
11 years or more	10	59	-	-	-	-
**Occupational or professional involvement in CMSP care†,‡**						
Clinical treatment of patients with CMSP	9	53	-	-	-	-
Teaching students about CMSP care	5	29	-	-	-	-
Teaching healthcare practitioners about CMSP care	3	18	-	-	-	-
Development of clinical practice guidelines	3	18	-	-	-	-
Research about CMSP	2	12	-	-	-	-
Implementation research	1	6	-	-	-	-
Policy development	1	6	-	-	-	-
Public healthcare sector	7	41	-	-	-	-
Private healthcare sector	5	29	-	-	-	-
Practice management	3	18	-	-	-	-

CMSP, chronic musculoskeletal pain.

† , data only available for round 1;

‡ , multiple options could be selected.

Out of the original core set of 43 recommendations, 36 were endorsed (a median score of 1 or 2 and an IQR of 1 or less) during Delphi round one and seven were not endorsed. Table 3 presents the recommendations for which consensus was achieved in the first Delphi round, which indicated recommendations that could be adopted for the SA context. Table 4 shows the original and reformulated recommendations with no consensus or undecided ratings at the end of Delphi round one, and the results after discussion at the panel meeting and re-voting on these statements in the second Delphi round. Table 4 therefore indicates recommendations that needed to be adapted or contextualised to be acceptable or feasible for the SA context. However, two recommendations received no consensus, and one was undecided, after contextualisation.

**TABLE 3:** Endorsed recommendations, their median rating and interquartile range.

Topic	Sub-topic	Recommendation	SoBE for the intervention	Source guideline/s	Median	IQR
Approach to care	Patient-centredness	We recommend the use of a compassionate, patient-centred approach for the assessment and management of chronic musculoskeletal pain. This includes the exploration of the patient’s beliefs, knowledge and understanding of pain and pain management to influence outcomes positively	Good SoBE	RNAO^24^ SIGN^25^	1	0
	Shared decision-making and goal setting	We recommend collaborative decision-making, which includes identifying patient goals; developing a comprehensive and patient-specific pain management strategy that considers the age, gender, ethnic and cultural background; and spirituality of the patient	Good SoBE	ICSI^26^ RNAO^24^	1	0
	Interprofessional collaboration	We recommend interprofessional collaboration and the development of an individualised and comprehensive plan of care based on the biopsychosocial model for the effective assessment and management of chronic musculoskeletal pain	Good SoBE	ICSI^26^ RNAO^24^	1	0
Assessment	Holistic assessment	We recommend performing a holistic patient evaluation, which includes history, physical examination, functional status, psychosocial risk factors and contextual factors in the evaluation, diagnosis and management of patients with chronic musculoskeletal pain	Good SoBE	ICSI^26^ RNAO^24^ SIGN^25^	1	0
	Assessment instruments	We recommend the use of appropriate, validated assessment tools to establish functional and psychological status and quality of life	Good SoBE	ICSI^26^ RNAO^24^	1	0
	Diagnostic and imaging procedures	We recommend that clinicians provide relevant and appropriate information to the patient when referring for diagnostic and imaging procedures. The results should be explained to the patient to mitigate fear, activity restriction, maladaptive behaviours and requests for opioids	Good SoBE	ASIPP^27^	1	0
	Re-assessment	We recommend regular re-assessment of the physical, psychological and social domains of the patient to determine the person’s response to pain management interventions	Good SoBE	RNAO^24^	1	0
Classification of pain	Classification of pain	We recommend the classification of chronic pain according to the type of pain as neuropathic, nociceptive, inflammatory, and mechanical (or mixed picture) to guide management	Good SoBE	ICSI^26^ SIGN^25^	2	1
Education	Address concerns	We recommend that clinicians address the patient’s concerns and beliefs and teach the person, their family and caregivers about pain management strategies	Good SoBE	NOUGG^28^	1	1
	Brief education	We recommend that brief education be given to patients with chronic musculoskeletal pain to facilitate the continuation of work or occupation	Good SoBE	SIGN^25^	1	1
	Advice to stay active	We recommend advice to stay active in addition to exercise therapy for patients with chronic low back pain to minimise long-term disability	Good SoBE	SIGN^25^	1	0
	Education about analgesia	We recommend that the clinician educate patients about the risks and benefits of all medications and monitor and manage side effects	Good SoBE	ICSI^26^ RNAO^24^	1	1
	Pain neuroscience education	We suggest that the clinicians consider pain neuroscience education to assist the patient in understanding their condition, change their conception about pain and improve their ability to cope with pain	Limited SoBE	Added by the panel, based on Wood et al.^29^	1	0
Physical therapy	Manual therapy	We recommend manual therapy, integrated with other interdisciplinary treatments for the short-term relief of chronic pain	Good SoBE	ICSI^26^ SIGN^25^	1	0
	Manual therapy and exercise	We recommend manual therapy in combination with exercise for the treatment of patients with chronic neck pain	Good SoBE	SIGN^25^	1	0
	Exercise	We recommend exercise and exercise therapies in the management of patients with chronic pain	Good SoBE	ICSI^26^ SIGN^25^	1	0
	Exercise adherence	We recommend the following approaches to improve adherence to exercise: supervised exercise sessions; individualised exercises in group settings; provision of a combined group and home exercise programme with the addition of supplementary material	Good SoBE	ICSI^26^ SIGN^25^	1	0
Electrotherapy	Transcutaneous electrical nerve stimulation (TENS)	We recommend TENS for the relief of chronic pain. Low or high-frequency TENS can be used	Good SoBE	ICSI^26^ SIGN^25^	2	0.5
Complementary therapy	Acupuncture	We recommend acupuncture for the short-term relief of pain in patients with certain pain conditions, such as chronic low back pain or osteoarthritis	Good SoBE	ICSI^26^ SIGN^25^	2	0.25
Psychological therapy	Identification of psychological comorbidities	We recommend that clinicians identify, manage and monitor comorbid psychological conditions such as depression in patients with chronic pain	Good SoBE	ICSI^26^	1	0
	Refer to psychologist	We suggest that clinicians consider assessing and addressing any concerns the patient may have about referral for psychological assessment by indicating that the approach to pain management is holistic. Explain the involvement of a psychologist to enhance coping skills	Limited SoBE	SIGN^25^	1	1
	Operant behavioural therapy	We recommend that clinicians be aware that the clinical environment and their own behaviour influence their responses and can impact patients’ responses	Good SoBE	SIGN^25^	1	1
	Cognitive behavioural therapy	We recommend cognitive behavioural therapy for functional restoration and reduction of pain in patients with chronic pain	Good SoBE	ICSI^26^ SIGN^25^ RNAO^24^	1	1
	Respondent behavioural therapy	We recommend progressive relaxation or electro-myographic biofeedback for the treatment of patients with chronic pain	Good SoBE	SIGN^25^	2	1
Pharmacological management	Analgesic review	We suggest that the clinician consider assessing a patient with chronic musculoskeletal pain using analgesics at least annually. More frequent review is necessary if medication is changed, or if the pain and underlying comorbidities alter	Limited SoBE	SIGN^25^	1	1
	Paracetamol	We recommend paracetamol, alone and in combination with NSAIDs and non-pharmacological treatments in the management of chronic musculoskeletal pain, such as hip or knee osteoarthritis	Good SoBE	ICSI^26^ SIGN^25^	1.5	1
	Oral NSAIDs	We recommend NSAIDs in the short term for chronic musculoskeletal pain such as chronic non-specific LBP and arthritis pain	Good SoBE	ICSI^26^ SIGN^25^	2	1
	NSAIDs risks	We recommend that clinicians consider cardiovascular, gastrointestinal and renal risks when prescribing NSAIDs, especially for older adults	Good SoBE	ICSI^26^ SIGN^25^	1	0
	Informed consent for opioids	We recommend that clinicians obtain informed consent before starting opioid therapy by advising the patient about potential benefits and risks	Good SoBE	Chou et al.^30^ NOUGG^28^ SIGN^25^	1	1
	Opioid risk assessment	We recommend that clinicians use a validated tool to do a risk assessment prior to prescribing opioids	Good SoBE	NOUGG^28^ SIGN^25^ ASIPP^27^	1	1
	Opioid therapy	We recommend opioid therapy for patients with moderate to severe chronic musculoskeletal pain (such as chronic low back pain or arthritis). Careful patient selection and regular review are required. Therapeutic benefits need to outweigh potential harms	Good SoBE	Chou et al.^30^ ASIPP^27^ ICSI^26^ NOUGG^28^ SIGN^25^	2	1
	Antidepressant therapy	We recommend antidepressant therapy for the treatment of patients with chronic pain and concomitant depression	Good SoBE	SIGN^25^	1	1
	Antidepressant therapy review	We suggest that the clinician consider reviewing patients with chronic musculoskeletal pain using antidepressants regularly to assess the on-going need for antidepressants and to ensure that the benefits outweigh the risks	Limited SoBE	SIGN^25^	1	0
	Muscle relaxants	We do not recommend the chronic use of muscle relaxants	Insufficient SoBE	ICSI^26^	1	1
	Anticonvulsants	We recommend the use of pregabalin for pain management in fibromyalgia	Good SoBE	SIGN^25^	1.5	1
Multidisciplinary management	Multidisciplinary pain management programme	We recommend referral to a multidisciplinary pain management programme for patients with chronic pain	Good SoBE	SIGN^25^	1	1
Specialist referral	Pain management specialist	We recommend referral to a pain management specialist when there is failure to achieve treatment goals; chronic pain is poorly controlled; there is significant distress and/or where interventional management or assessment is considered	Good SoBE	SIGN	1	1
Self-management	Self-management	We recommend self-management strategies and resources to be provided with other therapies in the treatment of patients with chronic pain to ensure active patient participation during early management as well as part of a long-term management	Good SoBE	ICSI^26^ SIGN^25^	1	0

*Source*: Adapted from Ernstzen DV, Louw QA. Synthesis of clinical practice guideline recommendations for the primary health care of chronic musculoskeletal pain. J Eval Clin Pract. 2022;28(3):454–467. https://doi.org/10.1111/jep.13644.

EML PHC SA, The Standard Treatment Guidelines and Essential Medicines List for Primary Health Care in SA;^31^ ASIPP, American Society of Interventional Pain Physicians; ICSI, Institute for Clinical Systems Improvement; IQR, interquartile range; LBP, low back pain; NOUGG, National Opioid Use Guideline Group; NSAIDs, non-steroidal anti-inflammatory drugs; RNAO, Registered Nurses’ Association of Ontario; SIGN, Scottish Intercollegiate Guidelines Network; SoBE, strength of the body of evidence; TENS, trans-electrical nerve stimulation.

Note: Median rating scale: strongly agree (1), partly agree (2), undecided (3), partly disagree (4) and strongly disagree (5). Consensus is an IQR of 1 or less^17^ and please see the full reference list of the article, Ernstzen DV, Parker R, Ras T, Von Pressentin K, Louw, QA. Clinical recommendations for chronic musculoskeletal pain in South African primary health care. Afr J Prm Health Care Fam Med. 2023;15(1), a3929. https://doi.org/10.4102/phcfm.v15i1.3929, for more information.

**TABLE 4:** Recommendations not endorsed in Delphi round one and results of Delphi round two.

Topic	Delphi round 1						Delphi round 2			
	Statement	Median	IQR	SoBE for the intervention	Source guidelines	Adapted or contextualised statements	Context point	Median	IQR
Tricyclic antidepressants	We recommend that tricyclic antidepressants should not be used for the management of chronic LBP	4	1.5	Good SoBE Adapted	SIGN^25^ Panel adapted recommendation based on Vos et al.^32^	We suggest that the clinician consider tricyclic antidepressants for the management of chronic LBP and concomitant depression	Current CPGs do not advocate tricyclic antidepressants for the management of chronic LBP. However, the burden of depression in the African context needs to be considered. A thorough evaluation of health status is warranted to identify signs and symptoms of depression. The dosage is dependent on numerous factors which can be sourced in the EML PHC SA	2	0.25
Amitriptyline	We recommend amitriptyline for the treatment of patients with fibromyalgia	2	1.25	Good SoBE	SIGN^25^	We recommend the tricyclic antidepressant amitriptyline for the treatment of patients with fibromyalgia	Amitriptyline is recommended as an adjuvant for the management of CMSP in the EML PHC SA. The dosage is dependent on numerous factors and a thorough evaluation of health status is warranted	2	1
Selective serotonin re-uptake inhibitor	We recommend the selective serotonin re-uptake inhibitor, fluoxetine for the treatment of pain and depression in patients with fibromyalgia	2	1.5	Good SoBE	SIGN^25^	We recommend the selective serotonin re-uptake inhibitor, fluoxetine for the treatment of pain and depression in patients with fibromyalgia	Fluoxetine is included in the EML PHC SA for the treatment of major depression	2	0.25
Serotonin and norepinephrine re-uptake inhibitor	We recommend the serotonin and norepinephrine re-uptake inhibitor, duloxetine for the treatment of patients with fibromyalgia or osteoarthritis	2.5	1	Good SoBE	ICSI^26^ SIGN^25^	We recommend serotonin and norepinephrine re-uptake inhibitor, duloxetine (where available) for the treatment of patients with fibromyalgia or osteoarthritis	Research studies about SNRIs for pain focused on duloxetine. Cost and policy factors in SA limit its use in PHC. Duloxetine is not included in the EML PHC SA	2	0.75
Topical NSAIDs	We recommend topical NSAIDs for the treatment of patients with chronic pain from musculoskeletal conditions.	3	1	Good SoBE	ICSI^26^ SIGN^25^	We suggest that the clinician consider topical NSAIDs for the treatment of inflammatory pain in patients with chronic musculoskeletal pain	The side effects of topical NSAIDs are like those of oral NSAIDs (albeit fewer).^33^ Oral and topical NSAIDs have a similar mechanism of action and should not be prescribed at the same time	2	1.25
Topical rubefacients	We recommend topical rubefacients for the treatment of pain in patients with musculoskeletal conditions if other pharmacological therapies have been ineffective.	3	1	Good SoBE	SIGN^25^ Panel adapted based on Derry et al.^34^	We do not recommend topical rubefacients for the treatment of pain in patients with musculoskeletal conditions.	The availability and cost of these creams limit its use in SA. The research base supports the use of capsaicin creams; however, these expensive creams are not readily available in SA. The use of salicylate creams is not supported by evidence^34^	2	2
LLLT	We recommend LLLT as a treatment option for patients with chronic LBP	3	1	Good SoBE	SIGN^25^	We suggest that the clinician consider LLLT as a treatment option for patients with chronic LBP	Consider the cost of apparatus, availability in the context, safety with application and training required	3	0.25

*Source:* Adapted from Ernstzen DV, Louw QA. Synthesis of clinical practice guideline recommendations for the primary health care of chronic musculoskeletal pain. J Eval Clin Pract. 2022;28(3):454–467. https://doi.org/10.1111/jep.13644

EML PHC SA, The Standard Treatment Guidelines and Essential Medicines List for Primary Health Care in SA;^31^ ICSI, Institute for Clinical Systems Improvement; IQR, interquartile range; LLLT, low-level laser therapy; NSAIDs, non-steroidal anti-inflammatory drugs; SA, South Africa; SIGN, Scottish Intercollegiate Guidelines Network; SNRI, serotonin and norepinephrine re-uptake inhibitors; SoBE, strength of the body of evidence; LBP, low back pain; CPGs, clinical practice guidelines; CMSP, chronic musculoskeletal pain.

Note: Median rating scale: strongly agree (1), partly agree (2), undecided (3), partly disagree (4) and strongly disagree (5); Consensus is an IQR of 1 or less^17^ and please see the full reference list of the article, Ernstzen DV, Parker R, Ras T, Von Pressentin K, Louw, QA. Clinical recommendations for chronic musculoskeletal pain in South African primary health care. Afr J Prm Health Care Fam Med. 2023;15(1), a3929. https://doi.org/10.4102/phcfm.v15i1.3929, for more information.

The seven recommendations that were not endorsed in the first Delphi round and therefore had uncertain applicability and feasibility to the SA PHC sector comprised six recommendations about pharmacological management for the prescription of topical non-steroidal anti-inflammatory drugs (NSAIDs), topical rubefacients and antidepressants for chronic pain conditions. Low-level laser therapy (LLLT) was the only non-pharmacological intervention with uncertain applicability and feasibility for the SA context. Although the recommendations for topical NSAIDs and rubefacients were adapted, they were not endorsed in Delphi round two because of their limited availability and the risk of side effects. The recommendations for tricyclic antidepressants for the management of chronic low back pain (LBP) were amended by the panel.

After two Delphi rounds, the panel reached a consensus on 41 recommendations, of which a recommendation on pain neuroscience education was added and endorsed by the panel. All recommendations, endorsed in round two, as well as six in round one, were endorsed as ‘partly agree’ indicating that uncertainty about these remain, motivating the need for further investigation.

Table 5 provides a summary of the contextual determinants listed by participants listed in the online Delphi rounds.

**TABLE 5:** Summary of contextual information provided by the participants in online Delphi rounds.

Criterion	Contextual determinants that will influence the implementation of recommendations
Broader or external context	Evidence base is important for uptake of recommendations
Organisational context	Human resources component: Access to the healthcare providers required to implement recommendations Time constraints influence implementability Availability of medicines in the local context Availability of equipment Affordability of the interventions
Practice method	Holistic assessment is important Healthy lifestyle initiatives are key components Safety considerations for each modality should be considered Careful consideration of dosages is indicated Optimise referral patterns Interdisciplinary collaboration involved
Intervention context	Clarification of terms indicated in the guidance Limited access to some of the interventions in the local context
Patient and carer context	Involvement of family in education about pain Patient-centredness and patients’ rights are vital in the process Use narratives and language that are locally applicable and culturally appropriate

## Discussion

**FIGURE 1:**
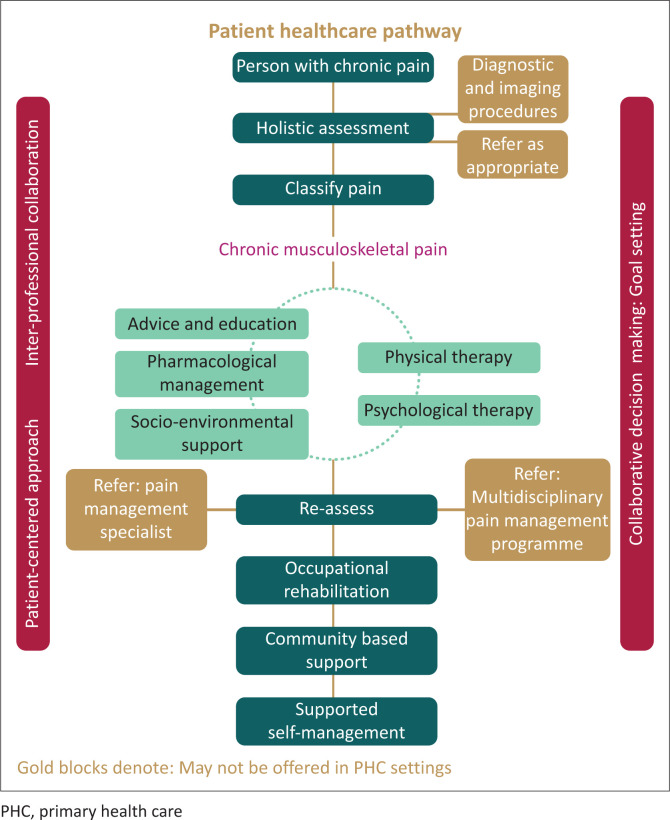
Primary health care pathway for a person with chronic musculoskeletal pain.

This study provides information on the acceptability and feasibility of evidence-based CPG recommendations for the multimodal care of CMSP in SA PHC settings. Interdisciplinary care and the involvement of nurses, medical practitioners, physiotherapists, occupational therapists, mental health practitioners, social workers and patient participation are emphasised to achieve therapeutic goals (Figure 1). We followed a structured and rigorous decision-making process via the participation of a panel of local experts who considered the evidence and context-specific circumstances for the applicability and feasibility of the recommendations. The study’s findings may be useful for decision-making regarding CMSP, when considering PHC interventions in the context of the NHI in SA. The endorsed recommendations indicate the aspects that may be prioritised to address in policy and practice to address the burden of CMSP in this context; and they provide clear direction to clinicians regarding interventions for CMSP and their underpinning body of evidence. The participants identified several contextual factors (Table 5) that may impact the implementability of the recommendations in practice. These contextual factors included organisation of health services at the primary care level, human resource capacity, practice patterns, access to care, intervention determinants, availability of equipment and patient-specific factors. Although certain recommendations were endorsed as applicable and feasible for the SA context, they may not be readily implementable without a change in contextual conditions, particularly in remote or rural communities. The implementation of the endorsed recommendations will require strategic prioritisation and a multisectoral implementation plan.

The recommendations support a person-centred approach to care and collaborative decision-making with the patient and the multidisciplinary team. The main outcome of the collaboration is the formation of a therapeutic alliance between the healthcare provider and the patient.^35^ With the given foundation, the healthcare provider can identify the patient’s priority concerns about CMSP, while considering the individual patient’s cultural beliefs, previous experience, values and beliefs regarding CMSP, to guide management and assessment decisions and to optimise the impact of the proposed interventions.^35^ In SA, with its diverse culture and language, the focus on the patient’s beliefs regarding pain is important, because the meaning and expression of pain is influenced by culture.^36^ Despite the importance of patient-centred care, the panel raised some concerns regarding human resource constraints, limited access to healthcare providers and limited available consultation time in PHC. The given contextual impediments are confirmed by existing literature^8,37^ and pose a threat to the approach to care as well as the implementation of several assessment and management options.

The multidimensional impact of CMSP requires holistic assessment. The panel agreed that the value of diagnostic imaging in chronic pain conditions should be carefully considered. This notion is supported by findings from a systematic review that negative diagnostic tests do not necessarily decrease the patient’s fear or concern about pain.^38^ However, investigations should be requested if requirements are met^39^ (i.e. serious pathology suspected, unsatisfactory response to care and unexplained progression of clinical picture) and when treatment decisions may alter based on the results. There still appears to be some uncertainty (albeit consensus) regarding the use of a mechanism-based classification of pain as nociceptive, neuropathic or nociplastic, as advocated in the ICD-11.^3^ The uptake of the classification approach in practice can be optimised using validated outcome measures, and it has several advantages for communication between healthcare providers.^3^ The panel agreed that validated outcome measures are valuable for assessing the impact of pain, as well as the quality of life and functional ability. The applicability of outcome measures may be influenced by their availability in different languages spoken in the different regions of SA, as well as their appropriateness for lifestyles, values, socio-economic factors and cultural beliefs in the intended target group. There is thus an impetus to develop a set of outcome measures that is contextually relevant for the local PHC context to assess the emotional, psychological, functional occupational and social impact of CMSP. Because the experience of chronic pain is influenced by social and cultural factors.^36^

Consistent with a multimodal approach to CMSP care, the panel recognised the importance of physical therapy, psychological therapy and socio-environmental support as a core first-line management for CMSP. Multidisciplinary management is important because of the close relationship between chronic pain, physical activity, psychological distress and the social determinants of health.^3,5,40^ A multidisciplinary healthcare professional should therefore be involved in CMSP care to strengthen the person-centred, biopsychosocial approach. While these recommendations are supported by evidence, the panel emphasised that access to rehabilitation and mental health professionals is constrained in the SA PHC context, when compared with high-income health systems, because of their relative scarcity.^8,37,41^ Mental health conditions and the reported lack of access to mental healthcare are an international concern. In addition, physical inactivity is a risk factor for the development of common mental disorders, emphasising the need for innovative solutions to address the interaction between chronic pain, physical activity and mental well-being.^5^ Therefore, there is an impetus to support SA PHC healthcare systems and organisations to enable interdisciplinary and intersectoral collaboration.^8^ The scarcity of multidisciplinary pain management programmes and pain management specialists in the SA context emphasises the need for interdisciplinary collaboration in the PHC context to achieve therapeutic goals.

Patient empowerment for self-management within a supportive environment is a key outcome of CMSP care. Empowerment towards self-management should commence early in the patient management pathway. The various recommendations focussing on patient education support this notion. Chronic musculoskeletal pain, because of its persistent nature, requires ongoing and supported self-management.^42^ Patient education can lead to increased understanding of the condition, enhanced healthcare literacy, behaviour change and adherence.^43,44^ However, education needs to be contextually relevant and take culture and health literacy into account aid patients’ understanding of CMSP as emphasised by the panel by published literature.^5,36^ Patient and family education requires a time investment, which was listed as an important need. Empowering and supportive environments can be achieved via community-based support programmes, to maintain gains made in the formal therapeutic environment,^43^ through peer support and public involvement.^42^ In the SA context, the Western Cape on Wellness (WoW)^45^ programme is an example of such a community-based support initiative.

Several factors influenced the endorsement of recommendations related to the pharmacological management of CMSP. The prescription of analgesics should be based on the step-wise approach and within the guidance of the SA Essential Medicines List for PHC (EML PHC).^46^ The endorsement for the use of opioids for moderate to severe CMSP came with several caveat recommendations, comprising careful patient selection, risk assessment and informed consent. The long-term use of opioids remains controversial, and the WHO released a statement cautioning on its use and advising further research in this area.^47^ The cautions regarding opioid prescription are supported by research because of associated side effects and the quality of the body of evidence on the topic. A Cochrane review^48^ found positive effects for pain and function in chronic LBP; however, the studies were of short duration, with limited data on long-term effects, and moderate to very low quality of evidence. Another Cochrane review^49^ concluded that there was not enough evidence to support the long-term use of tramadol alone, or tramadol in combination with paracetamol, for pain or functional improvements associated with osteoarthritis. These authors also reported a high incidence of adverse effects attributed to tramadol (nausea, dizziness and tiredness), which resulted in high attrition rates in the studies.^49^ For this study, it is acknowledged that most of the prescribers on the panel were based in public healthcare with opioid access limited to tramadol and morphine. There was a lack of representation of prescribers from the private healthcare sector, who have access to a wider variety of opioids for prescription. Therefore, the use of opioids in the management of CMSP should be carefully considered, based on the risk of addiction, risk of adverse effects and questionable long-term efficacy.

The recommendations regarding the anti-depressant drugs amitriptyline, fluoxetine and duloxetine were the focus of discussion during the consensus meeting in the study because of uncertainties that existed about them. The use of antidepressant medication in CMSP is an important consideration because depression is a common co-morbidity with CMSP^5^ and depression is one of the leading causes of years lived with disability in sub-Saharan Africa.^32^ Adaptations were made on the phrasing of these recommendations, based on information from the EML PHC^31^ and the burden of disease. For example, the recommendation on tricyclic antidepressants for the management of chronic LBP was contextualised based on the value judgement of the panel and considering the burden of disease in SA. In addition, Su et al.^50^ found that antidepressant therapy can improve outcomes of chronic pain even in the absence of clinical depression. It is acknowledged that, at the time of the study, the EML PHC^31^ was used, while the EML PHC^46^ is currently available. In summary, practical and policy implementation factors influenced participants’ choices for recommendations on pharmacological management, namely medication inclusion in the EML and availability in the PHC sector.

Certain recommendations were not endorsed because of concerns about their availability in the intended context, cost, safety and efficacy. The use of topical NSAIDs was not endorsed based on the risks associated with using oral and topical NSAIDs concurrently. A systematic review of the efficacy of topical NSAIDs concluded that they can be effective in the relief of osteoarthritis pain.^33^ It is therefore acknowledged that this recommendation might have benefitted from another Delphi round in which stable scoring might have been reached.^17^ The panel were undecided as to whether LLLT is applicable in the intended context in view of its limited availability, high cost and training required. The availability and access to electrotherapy equipment may have played a role in the decision-making, as acknowledged by the panel. There is also limited evidence to support or refute the effectiveness of LLLT for the treatment of LBP (subacute or chronic).^51^ The heterogeneity of the populations, interventions and comparison groups in the LLLT review provided insufficient data to draw firm conclusions. These findings indicate the need for higher-quality randomised controlled trials on the topic.

Based on the findings of the study, a patient pathway was synthesised to offer a visual overview of the recommendations and different decision-making points in a patient’s journey through the PHC sector. The discussion from the consensus meeting indicated that for the holistic management of people with chronic pain, additional interventions need to be considered for the local context, which had not been listed in the original CPGs. These additional recommendations form part of the patient pathway and are summarised in Table 6.

**TABLE 6:** Additional interventions to consider for the local context.

Topic	Suggestion
Socio-environmental concerns	We suggest that the clinician identify social and environmental stressors influencing the patient with chronic pain and refer the patient as appropriate (e.g. to a social worker).^40,52^
Occupational rehabilitation	We suggest that clinicians consider referring patients with chronic musculoskeletal pain for vocational assessment and rehabilitation to facilitate return to work and to reduce work-related disability.^53^
Community support programme	We suggest that the clinician consider referring the patient to a community support group, to enhance the focus on supported self-management.^43,45^

Note: Please see the full reference list of the article, Ernstzen DV, Parker R, Ras T, Von Pressentin K, Louw, QA. Clinical recommendations for chronic musculoskeletal pain in South African primary health care. Afr J Prm Health Care Fam Med. 2023;15(1), a3929. https://doi.org/10.4102/phcfm.v15i1.3929, for more information.

### Strengths and limitations of the study

The strength of this study was the systematic method that was followed to evaluate and endorse the recommendations. Through the electronic Delphi survey, the multidisciplinary panel confidentially provided their opinions about the applicability and feasibility of the recommendations for the intended context. We believe that the panellists used their scientific and contextual knowledge, and clinical experience integrated with their own belief system to make decisions.^18^ The endorsement of the CPG recommendations and the identification of context factors highlighted uniquely SA circumstances and pragmatic considerations. The panel provided information on factors that may influence the translation of the recommendations in practice. A thorough analysis of the context factors and the development of a multilevel implementation plan is recommended to enhance the uptake of recommendations into practice. A future study will provide an in-depth analysis of contextual factors that may influence the implementation of these endorsed recommendations in the SA PHC context. Patients and potential end-users also provided input regarding the recommendations,^19^ and the results of these inputs will be available in future publications.

The following limitations need to be considered when interpreting the findings of this study. Despite the delay in the publication of information, an updated search identified only one CPG that could potentially have been included in the synthesis process. The study did not include an analysis of the level of stability over Delphi rounds.^17^ A stability analysis was not conducted because of the focus on the two consensus criteria (the IQR and the number of rounds) as well as pragmatic considerations (time and resources). We opted to conduct a consensus meeting to provide an opportunity for discussion of potentially challenging recommendations. The consensus meeting was held in-person, which constrained participation. An online meeting was not considered, as the meeting took place in the pre-coronavirus disease 2019 (COVID-19) era when online meetings were not the norm. The selection of panel members may have influenced consensus.^16^ Despite a range of professions being invited to participate, the panel for the consensus meeting was skewed towards women and rehabilitation professionals. Only half of the invited participants participated in the second round, which may have led to participation bias. A major shortcoming was that we were unable to recruit a pharmacologist to the panel. However, a pharmacologist externally reviewed the recommendations to aid in quality assurance. There was limited participation of private sector practitioners. Most participants were from the urban areas of the Western Cape, and there was a limited representation of rural practitioners. The findings can therefore only be generalised to the Western Cape setting. Further research is required regarding the feasibility of the recommendations in rural contexts. We therefore recommend alternative recruitment strategies for similar studies in future to ensure geographical representativeness and representation of diverse professionals in the expert panel.^18^ We did not distinguish between the concepts of applicability versus feasibility, which could have yielded useful information. Furthermore, we acknowledge that while consensus was the focus, the utility of the study findings is dependent on the characteristics of the people who participated in the consensus process. Consensus does not necessarily imply the correct judgement but simply that there is agreement among the panellists.^18^ Patients were not directly part of this process, although their perspectives were considered.^19^ Patients therefore provided input on the contextual factors that should be considered for the recommendations; however, they did not have the opportunity to vote. It is acknowledged that the study took place prior to the COVID-19 pandemic and that the healthcare system changes and updates to the EML^46^ are not reflected in this study.

## Conclusion

A set of evidence-based recommendations for the PHC of CMSP pain in SA was endorsed by a multidisciplinary panel of local experts. This study summarises these recommendations for the assessment and management of CMSP, comprising pharmacological and non-pharmacological management to enhance a multidisciplinary approach. Future research should explore the influence of other context-specific factors within health systems and communities, which were reported as adjunct recommendations in this study as they could influence the uptake of the recommendations into practice.
